# Extending Anxiety Detection from Multimodal Wearables in Controlled Conditions to Real-World Environments

**DOI:** 10.3390/s25041241

**Published:** 2025-02-18

**Authors:** Abdulrahman Alkurdi, Maxine He, Jonathan Cerna, Jean Clore, Richard Sowers, Elizabeth T. Hsiao-Wecksler, Manuel E. Hernandez

**Affiliations:** 1 Department of Mechanical Science & Engineering, University of Illinois Urbana-Champaign, Urbana, IL 61801, USA; alkurdi2@illinois.edu (A.A.); ethw@illinois.edu (E.T.H.-W.); 2 Neuroscience Program, University of Illinois Urbana-Champaign, Urbana, IL 61801, USA; maoyuan2@illinois.edu (M.H.); cerna3@illinois.edu (J.C.); 3 Department of Psychiatry and Behavioral Medicine, University of Illinois College of Medicine at Peoria, Peoria, IL 61805, USA; jayclore@uic.edu; 4 Department of Industrial & Enterprise Systems Engineering, University of Illinois Urbana-Champaign, Urbana, IL 61801, USA; r-sowers@illinois.edu; 5 Department of Biomedical and Translational Sciences, Carle Illinois College of Medicine, University of Illinois Urbana-Champaign, Urbana, IL 61801, USA; 6 Department of Health and Kinesiology, University of Illinois Urbana-Champaign, Urbana, IL 61801, USA; 7 Department of Bioengineering, University of Illinois Urbana-Champaign, Urbana, IL 61801, USA; 8 Beckman Institute, University of Illinois Urbana-Champaign, Urbana, IL 61801, USA

**Keywords:** anxiety, machine learning, wearable technology, transfer learning, multimodal

## Abstract

This study quantitatively evaluated whether and how machine learning (ML) models built by data from controlled conditions can fit real-world conditions. This study focused on feature-based models using wearable technology from real-world data collected from young adults, so as to provide insights into the models’ robustness and the specific challenges posed by diverse environmental noise. Feature-based models, particularly XGBoost and Decision Trees, demonstrated considerable resilience, maintaining higher accuracy and reliability across different noise levels. This investigation included an in-depth analysis of transfer learning, highlighting its potential and limitations in adapting models developed from standard datasets, like WESAD, to complex real-world scenarios. Moreover, this study analyzed the distributed feature importance across various physiological signals, such as electrodermal activity (EDA) and electrocardiography (ECG), considering their vulnerability to environmental factors. It was found that integrating multiple physiological data types could significantly enhance model robustness. The results underscored the need for a nuanced understanding of signal contributions to model efficacy, suggesting that feature-based models showed much promise in practical applications.

## 1. Introduction

Anxiety disorders are among the most prevalent mental health issues worldwide, affecting millions and significantly impacting the quality of life [1,2,3]. While traditional methods like self-reports and clinical interviews offer insight into symptoms of anxiety, they are limited by subjectivity and intermittency [4]. The advent of wearable technology presents new possibilities for the continuous, objective, and non-invasive monitoring of anxiety symptoms in real-world settings.

Prior work has demonstrated the potential of wearable devices for anxiety detection using various physiological signals, such as electrodermal activity (EDA), heart rate variability (HRV), and accelerometer data [5,6,7,8]. However, these studies are predominantly conducted in controlled laboratory settings, which may not accurately reflect realistic conditions. While wearable devices have been used to assess anxiety in social settings [9] and predict the occurrence of anxiety [10,11,12,13,14], further work remains on evaluating the generalizability of pre-trained anxiety classifiers on lab data to real-world data and the benefits of noise on classifier robustness.

A recent review highlighted the use of machine learning to detect anxiety using physiological signals [15]. Building on recent work [10,14], this study extends beyond the controlled laboratory setting, delving into real-world environments to assess the practicality and effectiveness of wearable technology for monitoring anxiety. Using the open-source Wearable Stress and Affect Detection (WESAD) dataset [8], which consists of multimodal wearable sensor data in different affective states, we used a subset of data to align with the wearable hardware from our study.

Traditional machine learning (ML) models are typically trained and evaluated using the same dataset. The use of a single dataset provides increased homogeneity and consistency in data characteristics for use in establishing a benchmark of model performance. Prior work examining anxiety prediction [10,11,12,13,14,16,17,18,19,20,21,22] has typically focused on the use of self-reported measures [10,11,13,20] but has more recently focused on the use of objective physiological signals under controlled conditions [12,14,16,18,19,22]. However, few studies have examined the impact of physiological and non-physiological noise on anxiety prediction [22]. Thus, the evaluation of an ML model’s effectiveness under real-world conditions may provide insights into the models’ robustness and the specific challenges posed by diverse environmental noise.

Transfer learning is an approach that leverages knowledge from a pre-trained model to address a related problem with limited labeled data [23,24]. By adapting the pre-trained model to the new task, transfer learning reduces the need for extensive training data and computational resources [25,26]. It is particularly beneficial when the target task has limited labeled data, is similar to the pre-trained model’s task, or when training from scratch is expensive or time-consuming [26]. Transfer learning has been successfully applied in various domains, including computer vision, natural language processing, and speech [26,27]. However, few studies have examined the robustness of anxiety detection ML models to new datasets [19], and when they have, they have primarily focused on the use of controlled conditions.

This study addresses these gaps by evaluating feature-based machine learning (ML) models under a variety of real-world conditions. Feature-based ML models leverage domain expertise by providing a basis for features to use, based on prior evidence. We placed significant emphasis on distributed feature importance across different physiological signals, considering their specific failure modes which may affect detection accuracy. For example, EDA signals can be disrupted in wet or humid environments, acceleration data may be unreliable during physical activity, and electrocardiogram (ECG) signals may be affected by conductivity issues, making them challenging to integrate into wearable devices. Blood volume pulse (BVP) signals are also susceptible to disruptions caused by gaps between the device and the skin. By investigating the robustness and reliability of these models in challenging environments, this study provides an evaluation of the most effective modalities and measures for use in real-world applications.

The overarching goal of this study was to quantitatively evaluate whether and how ML models for anxiety detection built by data from wearables in controlled conditions could be applied to real-world environments. By leveraging data from young adults that reflect a wide range of environmental conditions, we aimed to identify the most informative features and modalities for anxiety detection and evaluate the use of open-source data to pre-train an anxiety classifier for use in novel tasks and conditions.

## 2. Materials and Methods

**Figure 1:**
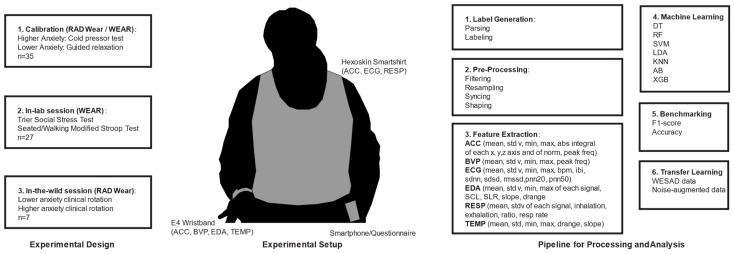
(**Left**) Experimental setup for in-lab and real-world data in RADWear and WEAR studies. (**Middle**) Experimental setup for data collection using wearable sensors, and (**Right**) pipeline for processing and analysis.

To evaluate the feasibility of using an open-source dataset for transfer learning and real-world data, we used custom Python (version 3.8) data processing and feature extraction scripts on both the WESAD dataset and real-world data (see Figure 1). Custom Python scripts are available in the following code repository: https://github.com/AbdulAlkurdi/anxietyFB (accessed on 3 February 2025). The WESAD dataset [8] was used as a basis for anxiety detection with physiological signals and the introduction of synthetic noise. The study involved 15 participants, with a gender distribution of 20% female, and the use of two wearable devices. The RespiBAN Professional (PLUX Wireless Biosignal S.A., Lisbon, Portugal) was used to record ECG, EDA, EMG, RESP, skin temperature (TEMP), and 3-axis acceleration (ACC) at 700 Hz, but was downsampled to 70 Hz for all modalities. The E4 Wristband (Empatica, Inc., Boston, MA, USA) was used to record BVP, EDA, TEMP, and ACC data at varying sampling rates. The study’s protocol included baseline (53%), amusement (17%), and stress (30%) conditions. Participant self-reports after each condition provided the ground truth, incorporating scales like Positive and Negative Affect Schedule (PANAS) [28], State-Trait Anxiety Inventory (STAI) [29], and Short Stress State Questionnaire (SSSQ) [30], as previously described.

### 2.1. Overview of Real-World Data

This study was designed to quantitatively evaluate whether and how ML models for anxiety detection built by data from wearables in controlled conditions could be applied to real-world environments using data from two ongoing studies. The WEAR study captures data from STEM-major undergraduate students. Participants included 12 males and 15 females with an average height of 161 ± 5 cm, weight of 67.5 ± 20 kg, and age of 23 ± 2.3 years. The RADWear study captures the demanding nature of medical education through data from third-year students on clinical rotations. Participants included 4 males and 5 females with an average height of 167 ± 7.5 cm, weight of 79.4 ± 27 kg, and age of 27.3 ± 2.3 years. Both studies have been approved by an institutional review board at either the University of Illinois College of Medicine at Peoria (IRB Protocol 1880297 for RADWear) or the University of Illinois Urbana-Champaign (IRB Protocol 21769 for WEAR).

For both studies, the E4 wristband and Hexoskin smart shirt (Carre Technologies, Montreal, QC, Canada) were used to collect data. The E4 wristband was utilized to collect various biophysiological signals including BVP, EDA, ACC, and skin temperature (TEMP). In parallel, the Hexoskin smart shirt recorded electrocardiogram (ECG), respiration (RESP), and 3-axis acceleration (ACC) data. For the Hexoskin smartshirt, ECG data were sampled at 256 Hz, while RESP and ACC data were sampled at 128 Hz and 64 Hz, respectively.

RADWear and WEAR involved a calibration protocol designed to establish baseline states for relaxation using a guided relaxation exercise and excitement/anxiety, captured during a cold pressor test in a lab setting. These initial sessions lasted about 30 min and aimed to provide reference points for trait anxiety levels, using the State-Trait Anxiety Inventory (STAI) X-2 questionnaire at the beginning and state anxiety changes after each task, using the STAI Y6.

In the WEAR study, participants underwent a comprehensive in-lab testing session lasting approximately four hours including the calibration session. After completing the calibration session, the participants performed an in-lab session that included a series of stress- and anxiety-inducing protocols: the Trier Social Stress Test (TSST), seated Stroop test, and walking Stroop test. These activities were selected to induce anxiety and serve as validated methods to establish baseline anxiety levels. WEAR participants began with an intake survey of trait anxiety (STAI X2) and completed follow-up questionnaires of state anxiety (STAI Y6) after each test to assess anxiety responses. Prior work has demonstrated significant differences in anxiety, based on the in-person tasks, and the ability to differentiate between different tasks using objective physiological signals [31].

In the RADWear study, following the calibration protocol, participants engaged in clinical rotations, and data were collected in real-world conditions. These “in-the-wild” data collection periods were extensive, consisting of two, two-week sessions (during work hours at least 5 days per week, lasting 6–8 h each day). For the current study, nine RADWear participants were included in the calibration sessions and seven in the in-the-wild data analysis, due to availability. However, future work remains necessary to increase the sample size and validate the effectiveness of the recorded data.

The current study datasets were organized into three subsets based on the similarity of the test conditions, including physical configuration and activities involved and environmental noise (Table 1): (1) RADWear and WEAR calibration sessions, where participants are restricted to seated conditions with a fixed room temperature and lighting conditions; (2) WEAR in-lab sessions, where participants are seated, standing, or walking in a controlled environment with a fixed room temperature and lighting conditions; and (3) RADWear in-the-wild datasets, where physical configuration and activity and environmental noise are variable and unconstrained. This selection of data subsets will inform how models perform under varying levels of environmental conditions that restrict participants’ motion and the potential for confounding factors.

### 2.2. Addressing Noise

Because of the RADWear and WEAR studies’ experimental protocols containing physical activity in both lab and natural settings, feature extraction presented more of a challenge. This was due to significant amounts of motion-artifact noise, which negatively affected the signal and the ability to extract features. Given the significant impact of noise on the feature extraction of heart rate signals, a feature extraction algorithm was developed, based on concepts from Malik et al. [32] and the Automatic Multiscale-based Peak Detection (AMPD) algorithm from Scholkmann et al. [33], which was able to achieve a 98% peak detection accuracy in ECG data. For EDA and ACC signals, we used a lowpass Butterworth filter with cut-off frequencies of 5 Hz and 13 Hz, respectively. Further details on data preprocessing and feature extraction are provided in recent work [22]. In summary, preprocessing steps included outlier removal, min–max normalization, feature extraction (see Figure 1), and handling of missing data, which were crucial for preparing RADWear and WEAR study data for effective machine learning analysis.

Feature extraction was performed on all available modalities from the E4 wristband and Hexoskin smart shirt, using features (Figure 1) that had shown anxiety detection robustness across a wide range of signal-to-noise ratios in XGB and DT models [22].

For the RADWear in-the-wild data, data loss due to excessive noise was partly resolved using imputation where sufficient data were available. If sufficient data in a segment were available, a mean imputation was employed to replace the missing points. If a significant portion of a segment was missing or too much noise existed, that segment was dropped.

### 2.3. Label Generation

**Figure 2:**
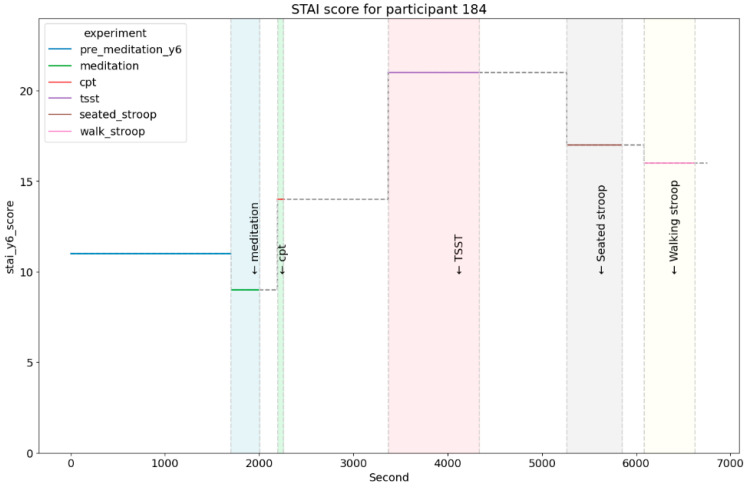
STAI score for one of the participants at the different test conditions experienced. The threshold considered to be indicative of the observation of anxiety is 11 for the STAI Y6. It can be observed that the meditation session reduced the anxiety level, while the cold pressor test (CPT) increased it. For this participant, the Trier Social Stress Test (TSST) seemed to be the highest cause of anxiety.

Using a cutoff of 11 on the STAI Y6 (Range 6–24), we identified data segments to correspond to either lower or higher anxiety, to serve as ground truth for the feature-based ML models. A representative sample of STAI scores across calibration and in-lab sessions is provided in Figure 2.

### 2.4. Class Balancing

Generally, to have effective classification outcomes from ML algorithms, datasets should be balanced between classes. Imbalanced datasets can lead to biased model performance, misleading performance metrics, limited generalization, and robustness. Thus, the use of balancing techniques to ensure fair and accurate machine learning models is of the utmost importance. In this study, the undersampling of the dominant class was utilized to reduce the imbalance. The laboratory-controlled datasets (RADWear + WEAR calibration, WEAR in-lab) had good balance. Due to the nature of the RADWear in-the-wild dataset, the anxious and non-anxious classes were imbalanced, with 80% of the data being labeled as not anxious. Undersampling was performed on the RADWear in-the-wild dataset to match that of the calibration and in-lab segments. Undersampling was performed by reducing the number of data points collected from the dominant class, which preserves the number of points of the minority class (Table 2).

### 2.5. Traditional Machine Learning

Based on recent findings on the most robust machine learning models in predicting anxiety from a noise-augmented WESAD dataset [22], feature-based models were employed. Feature-based models included the following: Decision Tree (DT), Random Forest (RF), Linear Discriminant Analysis (LDA), k-nearest neighbor (KNN), Adaboost (AB), Support Vector Machine (SVM), and XG Boost (XGB). All FB models were created using scikit-learn 1.2.1 ML library.

To train and evaluate each FB model, models were first initialized for each of the chosen algorithms, calculating performance metrics and feature importances directly during model evaluation. The training process involved setting up specific models, such as SVM, using the scikit-learn’s fit method. A 5-fold cross-validation method was utilized. The dataset was randomly divided into equal five test sets. For each of the test sets, the rest of the data were split into training (80%) and validation (20%) sets. For each of the architectures, 5-fold cross-validation and 5 training iterations were performed. Comparisons of these predictions with the actual labels facilitated the calculation of key performance metrics including accuracy and F1 score, averaged across the 5 folds.

Further, we generated a feature importance list for each model, which ranked the features by their influence on the model’s predictions. This provided critical insights into which variables significantly impacted the detection of anxiety. Detailed results for each model’s performance, along with the top seven influential features, were systematically documented to underscore the predictive capabilities and strengths of each model within the study.

### 2.6. Transfer Learning

**Figure 3:**
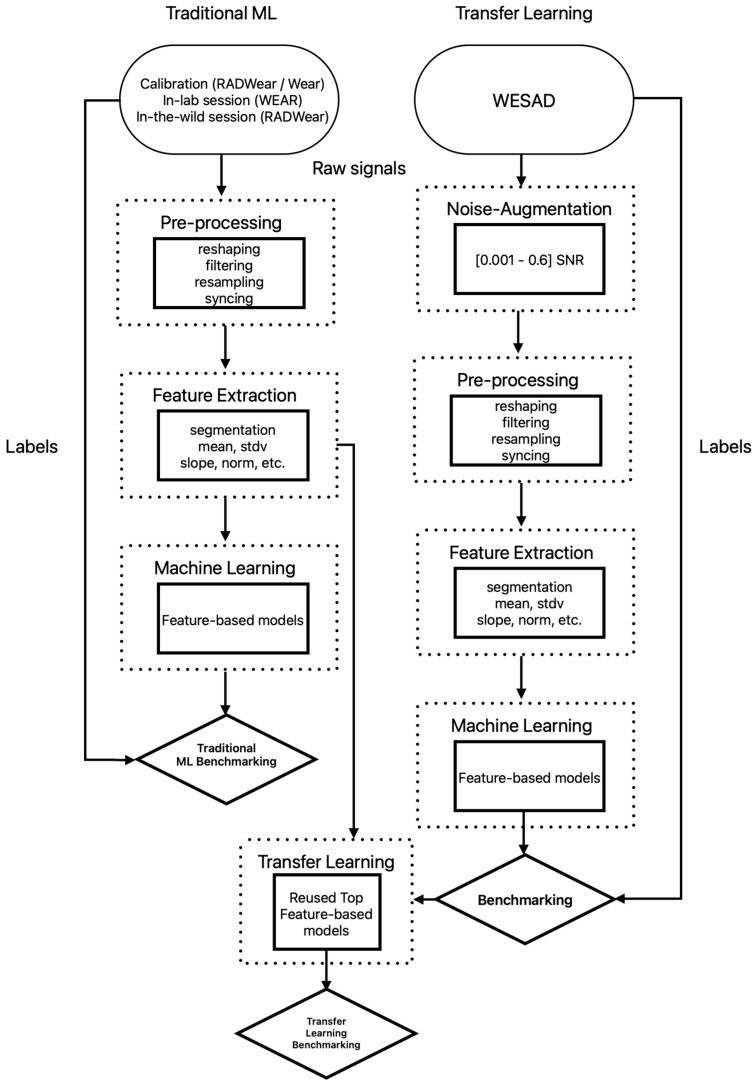
Flowchart outlining pipeline for processing and analyzing wearable sensor data from raw data collection to machine learning.

Afterwards, models that were trained using a subset of the WESAD dataset with and without noise augmentation that matched in modalities and were similar in locations were tested on the three subsets to test the validity of transfer learning for this application. Feature-based models were employed. DT, RF, LDA, KNN, AB, SVM, and XGB were utilized to test performance for transfer learning, using re-used models without additional fine-tuning. As seen in Figure 3, ML noise-augmented data were used to train feature-based models that were directly applied to the three subsets of RADWear and WEAR data.

Noise-augmented data derived from the WESAD dataset was used to assess the resilience of machine learning models to environmental disturbances. This approach was crucial for understanding how these models performed under simulated conditions that mimicked real-world noise, which is often encountered in daily activities and can significantly affect the accuracy of anxiety detection systems. Noise augmentation was carried out by adding Gaussian noise, ranging from 0.0001 to 0.6 signal-to-noise ratios (SNRs) from WESAD data.

By training models on both the original and noise-augmented WESAD data, we established a performance benchmark, examining how noise impacts model effectiveness and identifying which models maintain their predictive power despite increased noise levels. This process not only highlighted the robustness of certain models but also allowed for the optimization of these models to withstand typical real-world disruptions.

The transfer learning techniques applied here involved testing the models—initially trained on WESAD and its noise-augmented version—on the RADWear and WEAR data. The transition to testing on these studies, which feature real-life conditions and more complex environments than those simulated by noise augmentation, was facilitated by the preliminary insights gained from the noise impact analysis. This step ensured that the models not only generalized well across different types of input data but were also applicable in practical settings where environmental variability is the norm.

Overall, analyzing models with noise-augmented data enhances the relevance and applicability of these models for RADWear and WEAR data, where real-world conditions play a crucial role. It provided a solid foundation for understanding model performance in the face of unpredictable environmental factors, ensuring that the anxiety detection systems developed are both effective and reliable in varied real-world scenarios.

For transfer learning, models that have been tested on WESAD, and noise-augmented WESAD, were tested on the three subsets. The use of WESAD serves to establish a performance benchmark as a reference point. Since the models were initially trained and validated on this dataset, showcasing their performance on WESAD helps in understanding their efficiency and accuracy before they were tested under more variable conditions such as those provided by the RADWear and WEAR datasets. It is important to articulate that the WESAD dataset acts as a foundational dataset for training the models, whose performance metrics were crucial for establishing a comparative analysis.

The incorporation of noise-augmented data was a strategic decision to bolster the resilience of machine learning models against environmental noise, which is a common and disruptive element in real-world settings. The process of augmenting the WESAD dataset with noise simulates these realistic disturbances, thus enabling us to stress-test the models and prepare them for the unpredictable variances they would encounter in practical scenarios. By doing so, we aimed to ensure that the models retain their predictive accuracy even when faced with data that has been compromised or distorted by external factors. To assess the performance of each model evaluated at each subset, F_1_ score and accuracy were used to evaluate the models.

## 3. Results

### 3.1. Traditional ML Models

The traditional ML models refer to those tested and trained exclusively on each of the three data subsets, including the original WESAD dataset as a reference point for benchmarking. The analysis of the model performances with the RADWear and WEAR data (Table 3) highlighted the excellent performance of the XGB and DT models. Notably, the XGB model achieved a 0.99 accuracy and F1 score in the WESAD dataset [22]. This performance was maintained across the RADWear and WEAR calibration subsets, where XGB recorded a 0.95 accuracy and a 0.94 F1 score. Furthermore, even in the challenging conditions of the WEAR in-lab settings, XGB showed a robust performance with a 0.92 accuracy and a 0.90 F1 score. These results validate the effectiveness of traditional ML models in real-world scenarios, substantially outperforming the 50% accuracy expected of random guessing in binary classification.

Interestingly, the most predictive features varied across models and datasets. In Table 4, the performance metrics of various features across different models are presented, offering a detailed comparison of how different data types influence the effectiveness of the models. The “Weighted average” represents the aggregated influence of each feature across different models. This calculation was performed using a weighted mean, where each feature’s importance is squared within a model, summed across models, and then divided by the total sum of the feature importances. This calculation method emphasizes features that were consistently significant across various models, highlighting their predictive power in detecting anxiety. This approach particularly underscores the adaptability and critical role of specific features under conditions such as motion, where accelerometer data becomes increasingly significant.

### 3.2. Feature-Based Transfer Learning

**Figure 4:**
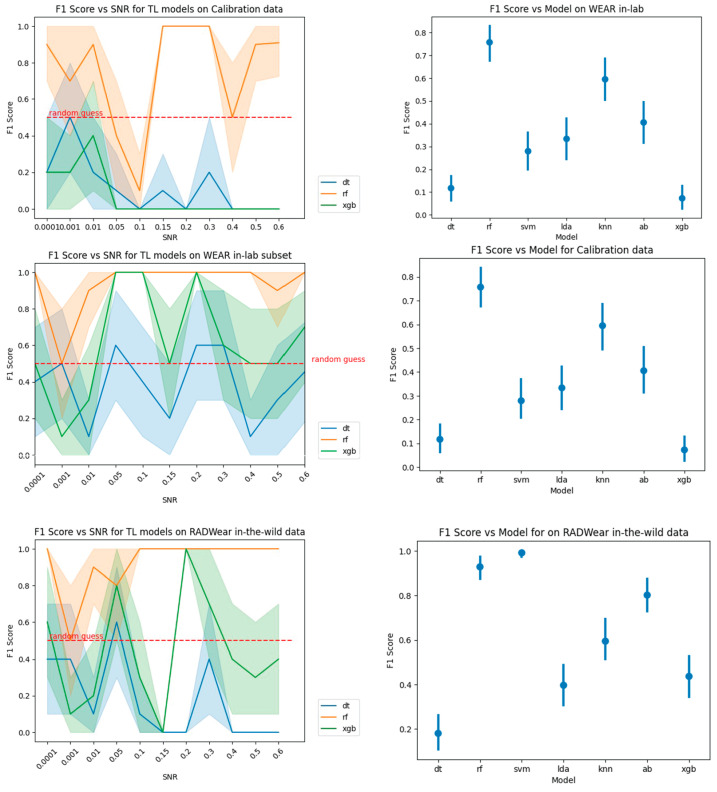
The F1 score of transfer learning models are shown with the left column showing F_1_ score for models trained at different noise levels at different signal-to-noise ratios (SNRs), shown in the x-axis. The right column shows F_1_ score for each of the tested models for all SNRs. F_1_ score, as tested on calibration data for WEAR and RADWear in the top row, WEAR in-lab sessions in the middle row, and on RADWear in-the-wild in the bottom row.

When evaluating the transfer learning performance (Figure 4 and Table 5), we discovered that the RF model excelled in the calibration and in-lab settings, while SVM achieved the best results for in-the-wild data. Intriguingly, models like XGB and DT, which previously outperformed in the baseline case, did not maintain their superiority in the transfer learning scenario. This finding highlights the importance of carefully selecting models based on the specific characteristics of the target dataset and the potential limitations of directly transferring models trained on one dataset to another.

In exploring the performance of transfer learning across our datasets, we identified a significant trend related to the distribution of feature importance weights in the models. Models exhibiting more evenly distributed feature importance weights demonstrated more successful adaptation when applied to new datasets, particularly when transitioning from controlled to more variable real-world conditions. For instance, models like LDA, which generally maintained balanced importance across features, adapted more effectively compared to those heavily reliant on one or two specific features, such as XGB. The latter showed reduced performance during transfer learning tasks, especially when applied to the in-the-wild subset of the WEAR dataset. This suggests that a more uniform distribution of feature weights might enhance a model’s adaptability, supporting better generalization across different experimental conditions and datasets. This finding underscores the importance of considering feature balance during model training for effective transfer learning applications.

## 4. Discussion

Our study contributes to the field of anxiety detection using wearables and machine learning by demonstrating the feasibility of developing robust models that can accurately detect anxiety in noisy environments and can be transferred from controlled laboratory conditions. The strong performance of our models, particularly XGB and DT, in both controlled and real-world settings, consistent with recent work in open-source datasets [22], underscores the potential for deploying such techniques in practical mental health monitoring applications.

In comparison to prior related work [14,19] examining physiological signals (Table 6), we find that the XGB and DT classifiers provide improved accuracy across all datasets examined in this study when using test data from the same dataset. Similarly, the F1 scores for the DT classifier demonstrated improved performance across all datasets except the in-the-wild data balanced to 41%. When examining transfer learning, by evaluating the use of training on one dataset and comparing to another, we found the best performance was provided by an RF classifier, consistent with prior work, which found a RF anxiety classifier to demonstrate robust performance when applying to a new dataset [19].

Prior work using the WESAD dataset has focused on stress detection using ML and deep learning approaches [8,34,35,36]. In addition, the in-lab portions of the WEAR study have been primarily focused on establishing the validity of physiological signal changes in stressful conditions [31]. However, further work remains to validate the in-the-wild portions of the RADWEAR study.

This study advances the existing literature in several ways. First, we conducted an analysis of feature importance, shedding light on the relative contributions of different physiological signals and derived features in anxiety detection, which could be potentially beneficial for individualized treatment planning. Second, we explored the applicability of transfer learning, highlighting the challenges and opportunities in adapting models trained on one dataset to another. Notably, it seems that models with feature importances close in weight perform better for transfer learning, while models with feature importances heavily weighted on one or two features, like XGB for calibration and in-the-wild, do not perform transfer learning as well.

The contrast in the identification of the most important modalities across each of the datasets suggests that physical activity and environmental noise significantly impact the most important information to classify anxiety. In contrast to prior work examining data from controlled environments [19,22], where EDA and BVP have been found to be among the most important modalities for anxiety classification, we found that ACC, TEMP, and ECG provided the most important modalities overall, as physical activity and environmental noise increased.

The study’s findings emphasize the importance of selecting features that were robust to various environmental factors and failure modes. Consequently, models that rely on a distributed feature importance, where multiple signals contribute to the detection of anxiety, were more likely to be resilient to individual signal failures. Future research should focus on identifying and engineering features that are robust to these challenges, enhancing the reliability of anxiety detection models in real-world settings.

However, our study is not without limitations. The sample sizes of the RADWear and WEAR datasets used in this study, while diverse, may not fully capture the entire spectrum of individual variability in anxiety responses. As those studies are ongoing, these datasets will expand, incorporating a broader range of participant demographics and clinical profiles, so as to better generalize new subjects. Additionally, while our feature-based models demonstrate promising performance, further validation in longitudinal studies and real-world deployments would be necessary to assess their long-term reliability and usefulness in clinical practice. Further, the high accuracies but lower F1 scores observed in some of the transfer learning suggest potential overfitting, which should be further examined in future work.

Developing accurate and robust anxiety detection models can pave the way for personalized mental health interventions that are delivered in real-time using wearable devices, potentially enhancing the effectiveness of current empirically supported therapy, improving the accuracy of self-report data, and providing “self-help” programs beyond common bibliotherapy, thereby reaching more rural populations where specialized clinicians may not be available. This could revolutionize the way we monitor and manage anxiety disorders, enabling early detection, timely support, and targeted treatments. However, translating these models into practical tools will require addressing challenges such as data privacy, user acceptance, and seamless integration with existing healthcare systems.

## 5. Conclusions

This study underscores the efficacy of feature-based models, particularly XGBoost and DT, in accurately detecting anxiety under both controlled conditions and real-world environments, and for models such as RF to transfer learning well from different datasets. The introduction of the RADWear and WEAR studies enhances our understanding of the robustness of these models amidst environmental noise and varied conditions. The analysis of feature importance and the implementation of transfer learning have significantly contributed to advancing the field of anxiety detection using wearable technology. Key findings emphasize the resilience of feature-based models and the critical role of precise feature selection in maintaining model accuracy across diverse settings. These insights not only validate the effectiveness of current methodologies but also underscore the potential of these models in practical mental health monitoring applications.

## Figures and Tables

**Table 1:** Noise factors considered for each subset of the datasets.

	Physical Configuration and Activity				Environmental Noise	
Dataset	Seated	Standing	Walking	Other	Fixed	Variable
WESAD						
Calibration (RADWear/WEAR)						
In-lab session (WEAR)						
In-the-wild session (RADWear)						

Note: Black color used to indicate physical configuration and activity or environmental noise condition in each dataset.

**Table 2:** Class distribution for each subset of the datasets.

Dataset	Not Anxious	Anxiety	Adjusted
WESAD	70%	30%	×
RADWear + WEAR Calibration	58%	42%	×
WEAR in-lab	59%	41%	×
RADWear in-the-wild	80.66%	19.34%	to 30% and 41%

**Table 3:** F1-score performance results of traditional ML models trained and tested on the same subset of the datasets.

Data	XGB		DT		LDA		RF	
	ACC	F_1_	ACC	F_1_	ACC	F_1_	ACC	F_1_
WESAD	0.99	0.99	0.99	0.99	0.93	0.92	0.91	0.89
RADWear + WEAR Calibration	0.95	0.94	0.91	0.9	0.82	0.8	0.88	0.86
WEAR in-lab	0.92	0.9	0.93	0.94	0.69	0.65	0.8	0.76
RADWear in-the-wild (balanced to 30%)	0.87	0.77	0.97	0.95	0.79	0.52	0.82	0.72
RADWear in-the-wild (balanced to 41%)	0.88	0.58	0.99	0.66	0.73	0.61	0.78	70
Random guess	0.5							

Note: Grey color used to indicate top performing classifier, based on accuracy (ACC) or F1 score (F_1_), for each dataset.

**Table 4:** Feature importance for traditional ML models on calibration, in-lab, and in-the-wild data.

	Modality	Modality Weighted	Feature	Feature Weighted	RF	DT	XGB
RADWear in-the-wild	ECG	0.13	ECG_min_	0.17	0.14	0.18	0.19
			ECG_std_	0.08	0.08		
			ECG_max_	0.06	0.06	0.04	0.07
	Resp	0.07	Resp_Exhal std_	0.07	0.07		
			Resp_mean_	0.07	0.07	0.09	0.04
			Resp_rate_	0.06	0.07		0.06
			Resp_I/E_	0.06	0.06		
			Resp_min_	0.08		0.08	0.08
	EDA	0.06	EDA_mean_	0.07		0.07	
			EDA_drange_	0.06			0.06
	BVP	0.04	BVP_peak freq_	0.04			0.04
	TEMP	0.11	TEMP_mean_	0.11		0.11	
	ACC	0.06	ACC_net w mean_	0.06		0.06	
RADWear + WEAR Calibration	EDA	0.1	EDA_mean_	0.12	0.06	0.17	0.05
			EDA_max_	0.05			0.05
	RESP	0.05	Resp_min_	0.07		0.07	
			Resp_mean_	0.05	0.05		0.05
			Resp_rate_	0.05		0.05	0.04
			Resp_Inhalmean_	0.04		0.03	0.04
	BVP	0.04	BVP_max_	0.04	0.04		
	ACC	0.13	ACC_net w std_	0.21	0.08	0.26	0.2
			ACC_net w mean_	0.04		0.04	
			ACC_x min_	0.06		0.06	
			ACC_x C std_	0.08	0.08		
			ACC _x min_	0.06	0.07		0.04
			ACC_x mean_	0.05	0.05		
WEAR in-lab	ECG	0.12	ECG_bpm_	0.08			0.08
			ECG_max_	0.13	0.14	0.14	0.12
			ECG_min_	0.07	0.07		
			ECG_std_	0.15	0.2	0.12	0.07
	EDA	0.05	EDA_max_	0.05		0.06	0.04
			EDA_mean_	0.05		0.05	
	RESP	0.06	Resp_mean_	0.07	0.08	0.09	0.05
			Resp_min_	0.04	0.04		0.05
	ACC	0.05	ACC_x mean_	0.05		0.06	0.05
			ACC_net w mean_	0.05	0.05		
	TEMP	0.14	TEMP_mean_	0.14	0.12	0.16	

Note: Grey color used to indicate most important modality and feature for each dataset and classifier.

**Table 5:** Performance results for transfer learning models on calibration, in-lab, and in-the-wild data.

Model	DT		RF		XGB	
	Accuracy	F_1_-Score	Accuracy	F_1_-Score	Accuracy	F_1_-Score
RADWear + WEAR Calibration	0.12	0.12	0.76	0.76	0.07	0.07
WEAR in-lab	0.18	0.39	0.93	0.94	0.44	0.61
RADWear in-the-wild	0.18	0.18	0.93	0.93	0.44	0.44
anxiety class balanced to 41%						

**Table 6:** Anxiety classification performance results of related works using physiological signals.

Authors	Dataset Used	Features	Psychological Measures	Physical Activity or Condition	Environmental Noise	Classification/Cut-Off	Performance (Accuracy)	Performance (F1)	TL Performance (Accuracy)	TL Performance (F1)
Henry et al., 2023 [19]	CASE: customized anxiety index from self-report arousal and valance scores	ECG and BVP	Anxiety index	Seated	Fixed	Anxiety State, using anxiety index with 0.5 cutoff	RF(BVP) = 0.752; SVM(ECG) = 0.763.	RF(BVP) = 0.759; SVM(ECG) = 0.770.	WESAD to CASE: SVM, RF, XGBoost, MLP, ResNet(BVP) = 0.501; XGBoost(ECG) = 0.605.	WESAD to CASE:FTTP(BVP) = 0.107; FTTP(ECG) = 0.554.
	WESAD: Study protocol design of baseline, amusement, stress	ECG and BVP	PANAS, STAI, SAM, and SSSQ	Seated/Standing	Fixed	Stress vs. Non-Stress using protocol label	SVM(BVP) = 0.745; SVM(ECG) = 0.811.	SVM(BVP) = 0.773; SVM(ECG) = 0.818.	CASE to WESAD: MLP(BVP) = 0.587; RF(ECG) = 0.657.	CASE to WESAD: SVM(BVP) = 0.694; FTT(ECG) = 0.694.
Perpetuini et al., 2020 [14]	Study protocol examined supine rest with and without video	BVP	STAI	Supine	Fixed	Anxiety State, using STAI with 40 cutoff	AUC GLM(BVP) = 0.88;	NA	NA	NA

## Data Availability

The raw data supporting the conclusions of this article will be made available by the authors, without undue reservation.

## References

[1] Celano C.M., Daunis D.J., Lokko H.N., Campbell K.A., Huffman J.C. (2016). Anxiety Disorders and Cardiovascular Disease. Curr. Psychiatry Rep..

[2] Critchley H.D. (2002). Study of the Stress Response: Role of Anxiety, Cortisol and DHEAs. Neuroscientist.

[3] Bär K.J., Critchley H. (2015). Autonomic Control. Brain Mapp. Encycl. Ref..

[4] Julian L.J. (2011). Measures of Anxiety. Arthritis Care.

[5] Healey J.A., Picard R.W. (2005). Detecting Stress during Real-World Driving Tasks Using Physiological Sensors. IEEE Trans. Intell. Transp. Syst..

[6] Elgendi M., Galli V., Ahmadizadeh C., Menon C. (2022). Dataset of Psychological Scales and Physiological Signals Collected for Anxiety Assessment Using a Portable Device. Data.

[7] Haouij N.E., Poggi J.M., Sevestre-Ghalila S., Ghozi R., Jadane M. (2018). Affective ROAD System and Database to Assess Driver’s Attention. Proceedings of the 33rd Annual ACM Symposium on Applied Computing.

[8] Schmidt P., Reiss A., Duerichen R., Laerhoven K. (2018). Van Introducing WeSAD, a Multimodal Dataset for Wearable Stress and Affect Detection. Proceedings of the 2018 International Conference on Multimodal Interaction.

[9] Shaukat-Jali R., van Zalk N., Boyle D.E. (2021). Detecting Subclinical Social Anxiety Using Physiological Data from a Wrist-Worn Wearable: Small-Scale Feasibility Study. JMIR Form. Res..

[10] Sanjay V.M., Ankith I., Dhanush G.C., Akshaya H.P., Malik J., Tejaswini B.N. Anxiety Prediction during Stressful Scenarios Using Machine Learning. Proceedings of the 2022 6th International Conference on Intelligent Computing and Control Systems, ICICCS 2022.

[11] Albagmi F.M., Alansari A., Shawan D.S.A., AlNujaidi H.Y., Olatunji S.O. (2022). Prediction of Generalized Anxiety Levels during the Covid-19 Pandemic: A Machine Learning-Based Modeling Approach. Inf. Inform. Med. Unlocked.

[12] Ramani B., Patel W., Solanki K. (2022). Stress Ocare: An Advance IoMT Based Physiological Data Analysis for Anxiety Status Prediction Using Cloud Computing. J. Discret. Math. Sci. Cryptogr..

[13] Srinath K.S., Kiran K., Pranavi S., Amrutha M., Shenoy P.D., Venugopal K.R. Prediction of Depression, Anxiety and Stress Levels Using Dass-42. Proceedings of the 2022 IEEE 7th International conference for Convergence in Technology, I2CT 2022.

[14] Perpetuini D., Chiarelli A.M., Cardone D., Filippini C., Rinella S., Massimino S., Bianco F., Bucciarelli V., Vinciguerra V., Fallica P. (2021). Prediction of State Anxiety by Machine Learning Applied to Photoplethysmography Data. PeerJ.

[15] Ancillon L., Elgendi M., Menon C. (2022). Machine Learning for Anxiety Detection Using Biosignals: A Review. Diagnostics.

[16] Sato M., Ishikawa W., Suzuki T., Matsumoto T., Tsujii T., Sakatani K. (2013). Bayesian STAI Anxiety Index Predictions Based on Prefrontal Cortex NIRS Data for the Resting State. Adv. Exp. Med. Biol..

[17] Baird A., Cummins N., Schnieder S., Krajewski J., Schuller B.W. An Evaluation of the Effect of Anxiety on Speech ? Computational Prediction of Anxiety from Sustained Vowels. Proceedings of the Annual Conference of the International Speech Communication Association, INTERSPEECH.

[18] Fukuda Y., Ishikawa W., Kanayama R., Matsumoto T., Takemura N., Sakatani K. (2014). Bayesian Prediction of Anxiety Level in Aged People at Rest Using 2-Channel NIRS Data from Prefrontal Cortex. Adv. Exp. Med. Biol..

[19] Henry J., Lloyd H., Turner M., Kendrick C. (2023). On the Robustness of Machine Learning Models for Stress and Anxiety Recognition from Heart Activity Signals. IEEE Sens. J..

[20] Xiong H., Berkovsky S., Romano M., Sharan R.V., Liu S., Coiera E., McLellan L.F. (2021). Prediction of Anxiety Disorders Using a Feature Ensemble Based Bayesian Neural Network. J. Biomed. Inf. Inform..

[21] Adheena M.A., Sindhu N., Jerritta S. (2018). Physiological Detection of Anxiety. Proceedings of the 2018 International Conference on Circuits and Systems in Digital Enterprise Technology (ICCSDET).

[22] Alkurdi A., Clore J., Sowers R., Hsiao-Wecksler E.T., Hernandez M.E. (2025). Resilience of Machine Learning Models in Anxiety Detection: Assessing the Impact of Gaussian Noise on Wearable Sensors. Appl. Sci..

[23] Pan S.J., Yang Q. (2010). A Survey on Transfer Learning. IEEE Trans. Knowl. Data Eng..

[24] Singh M., Prakash P., Kaur R., Sowers R., Brašić J.R., Hernandez M.E. (2023). A Deep Learning Approach for Automatic and Objective Grading of the Motor Impairment Severity in Parkinson’s Disease for Use in Tele-Assessments. Sensors.

[25] Weiss K., Khoshgoftaar T.M., Wang D.D. (2016). A Survey of Transfer Learning. J. Big Data.

[26] Zhuang F., Qi Z., Duan K., Xi D., Zhu Y., Zhu H., Xiong H., He Q. (2021). A Comprehensive Survey on Transfer Learning. Proc. IEEE.

[27] Shao L., Zhu F., Li X. (2015). Transfer Learning for Visual Categorization: A Survey. IEEE Trans. Neural Netw. Learn. Syst..

[28] Watson D., Clark L.A., Tellegen A. (1988). Development and Validation of Brief Measures of Positive and Negative Affect: The PANAS Scales. J. Pers. Soc. Psychol..

[29] Spielberger C.D., Gonzalez-Reigosa F., Martinez-Urrutia A., Natalicio L.F.S., Natalicio D.S. (1971). The State-Trait Anxiety Inventory. Interam. J. Psychol..

[30] Helton W.S. (2004). Validation of a Short Stress State Questionnaire. Proc. Hum. Human. Factors Ergon. Soc. Annu. Meet..

[31] He M., Cerna J., Alkurdi A., Dogan A., Zhao J., Clore J.L., Sowers R., Hsiao-Wecksler E.T., Hernandez M.E. Physical, Social and Cognitive Stressor Identification Using Electrocardiography-Derived Features and Machine Learning from a Wearable Device. Proceedings of the 2024 46th Annual International Conference of the IEEE Engineering in Medicine and Biology Society (EMBC).

[32] Malik M., Camm A.J., Bigger J.T., Breithardt G., Cerutti S., Cohen R.J., Coumel P., Fallen E.L., Kennedy H.L., Kleiger R.E. (1996). Heart Rate Variability: Standards of Measurement, Physiological Interpretation, and Clinical Use. Circulation.

[33] Scholkmann F., Boss J., Wolf M. (2012). An Efficient Algorithm for Automatic Peak Detection in Noisy Periodic and Quasi-Periodic Signals. Algorithms.

[34] Gil-Martin M., San-Segundo R., Mateos A., Ferreiros-Lopez J. (2022). Human Stress Detection with Wearable Sensors Using Convolutional Neural Networks. IEEE Aerosp. Electron. Syst. Mag..

[35] Ghosh S., Kim S., Ijaz M.F., Singh P.K., Mahmud M. (2022). Classification of Mental Stress from Wearable Physiological Sensors Using Image-Encoding-Based Deep Neural Network. Biosensors.

[36] Dziezyc M., Gjoreski M., Kazienko P., Saganowski S., Gams M. (2020). Can We Ditch Feature Engineering? End-to-End Deep Learning for Affect Recognition from Physiological Sensor Data. Sensors.

